# Blood Safety Status in WHO African Region Countries: Lessons Learnt from Mauritius

**DOI:** 10.1155/2017/1970479

**Published:** 2017-10-17

**Authors:** André Loua, Janaki Sonoo, Laurent Musango, Jean Baptiste Nikiema, Thomas Lapnet-Moustapha

**Affiliations:** ^1^WHO Regional Office for Africa, Brazzaville, Congo; ^2^National Blood Transfusion Service of Mauritius, Port Louis, Mauritius; ^3^WHO Country Office, Port Louis, Mauritius

## Abstract

In 2001, the WHO Office for Africa adopted a strategy for blood safety defining four targets. This paper describes the progress made by Mauritius in the implementation of this strategy. The blood safety indicators were collected and compared with the norms recommended by WHO. The country has formulated its blood policy and developed a strategic plan for its implementation since 2004. The total number of blood donations increased from 31,228 in 2002 to 43,742 in 2016, giving an annual blood collection rate evolving from 26.3 per 1000 inhabitants in 2002 to 34.2 per 1000 inhabitants in 2016. The percentage of voluntary donations rose from 60% to 82.5%. Since 2002, all the blood units collected have been tested for the mandatory infectious markers. The Blood Transfusion Service has been certified ISO2008-9001 and nucleic acid testing has been introduced. The preparation of blood components increased from 60% to 98.2%. The most transfused blood components were red cell concentrates, platelet concentrates, and fresh frozen plasma. In addition to transfusion activities, there were other departments performing antenatal serology, tissue typing, special investigations, and reagent preparation. Despite the progress made, some challenges remain, namely, legal framework and haemovigilance system. A regulatory system for blood needs to be established.

## 1. Introduction

Blood safety is a WHO global and regional priority. In May 1975, the World Health Assembly adopted resolution WHA28.72, urging Member States to promote the development of coordinated national blood transfusion services based on voluntary and nonremunerated blood donations (VNRBD), enact effective legislation governing the operation of these services, and take other necessary actions to protect and promote the health of blood donors and recipients of blood and blood products [1].

Pursuant to this resolution, the Regional Committee for Africa, in resolution AFR/RC44/R12 of 1994, urged Member States in the African Region to take urgent steps towards formulating blood safety policies, mobilizing resources for blood service infrastructure development in central and district hospitals, and setting goals and targets to achieve HIV-free blood transfusion in health care settings [2]. Furthermore, to improve the availability of safe blood products in all Member States, the Regional Office for Africa adopted a regional strategy for blood safety in September 2001 [3] whose main objectives were to (i) assist countries in setting up an effective system for the recruitment of low-risk donors, (ii) improve the safety of blood and blood products by implementing a quality assurance programme and mapping out effective strategies for the screening of blood for all transfusion-transmitted infections (TTIs), and (iii) promote the appropriate use of blood and blood products by clinicians. This strategy defined four targets to be met by each country, namely, (i) a blood safety situation analysis carried out in all Member States; (ii) a national blood policy developed and adopted by 75% of the countries in the Region; (iii) mandatory testing of all transfused blood units for the four disease markers: human immunodeficiency virus (HIV), hepatitis B virus (HBV), hepatitis C virus (HCV), and syphilis; and (iv) ensuring that at least 80% of blood donations in all countries in the Region come from VNRBD.

Mauritius is an Indian Ocean island state which lies about 2,000 kilometres off the southeast coast of the African continent and has an estimated resident population of 1,262,862 inhabitants. It has enjoyed a stable political and economic situation since independence in 1968 and has an upper middle-income economy, according to the World Bank in 2011. The country ranks high in terms of economic competitiveness, a friendly investment climate, good governance, and a free economy. Mauritius is performing well on the three health indicators commonly used to compare the health status of countries, namely, life expectancy at birth and the infant mortality and maternal mortality rates per thousand live births. Noncommunicable diseases (NCDs) such as diabetes and cardiovascular diseases are emerging and remain a major challenge for Mauritius, accounting for almost 80% of the disease burden with 85% of deaths [4]. The supply of blood in the country is crucial for certain medical cases like surgeries, cancer, anaemia treatments, renal dialysis, transfusions, and accident victims [5].

The objective of this article is to evaluate the implementation of the global and regional resolutions on blood availability and safety in Mauritius from 2002 to 2016, in order to measure progress made at the country level using specific indicators from the global database on blood safety (GDBS) and the targets of the regional strategy for blood safety; identify successes, constraints, and challenges; and define the priority actions to be undertaken in the coming years [6–8].

## 2. Materials and Methods

A first questionnaire based on the global blood safety (GDBS) indicators was used in Mauritius in 2002 to collect data. This questionnaire has been progressively updated and used to collect data from 2003 to 2013. The key indicators were related to facilities of blood transfusion services, blood policy, legislation governing transfusion, funding of blood safety, voluntary, nonremunerated blood donations, of blood components preparation (e.g., one whole blood donation can be used for the preparation of units of red cell concentrate, fresh frozen plasma, platelets, and cryoprecipitate), screening of blood donations for TTIs, guidelines on the rational use of blood and blood products, establishment of national haemovigilance systems, and so on.

An additional self-assessment questionnaire developed by the WHO Regional Office for Africa (WHO/AFRO) was used to collect data on the achievement of the targets of the regional strategy for blood safety from 2014 to 2016 [9].

Data collected from these questionnaires were compiled and analysed to show the trends. The survey reports of WHO/AFRO and annual activity reports of national blood transfusion service (NBTS) of Mauritius were used for the analysis propose [6–9]. The annual rate of blood units collected per 1,000 population was calculated using national census estimates [4], followed by a SWOT (strengths, weaknesses, opportunities, and treats) analysis. Priority actions were proposed to address challenges that have been identified in order to strengthen the national blood transfusion system.

## 3. Results

### 3.1. National Blood System Development, Organization, and Management

In order to implement the global and regional strategies for blood safety, in 2004 the country formulated and adopted a national blood policy that is currently being implemented. The blood policy, which was updated and is now known as the Mauritius Blood Service Act 2010, seeks to promote blood donation and ensure the safe and adequate supply of blood and blood products. However, the legislation framework for transfusion has not yet been developed and the regulatory system for blood and blood products had not been established. The priority actions identified were focused on (i) the identification of the service with national responsibility for provision of safe blood and blood products for transfusion in the country, including the responsibility for collection, testing, processing, and distribution of blood and blood products, and for liaison with health care facilities for appropriate clinical use of blood; (ii) the setting up of suitable infrastructure and facilities for blood transfusion services; (iii) the strengthening of capacity of blood services in human in adequate number of qualified and trained staff; (iv) the development of national standards and the implementation of quality system in blood transfusion services including information management system; and (v) the funding of blood safety.

The National Blood Transfusion Service (NBTS) of Mauritius operates under the Ministry of Health and Quality of Life (MoH&QL) and it is one of the many departments of the Central Health Laboratory and addresses the blood and blood component needs of all the public and private health care institutions in Mauritius. Four regional blood banks are in place within the regional hospitals and the maximum distance between them and the NBTS is 40 km that can be covered in less than an hour. Their main activities are limited to the collection of blood from blood donors and storage and blood delivery to hospitals.

A national quality assurance programme has been in place since the NBTS established national standards for the collection, testing, processing, storage, and distribution of blood and blood products and also participated in a national external quality assessment scheme (EQAS) for TTIs, laboratory screening, blood group serology, and compatibility testing. The score of performance of EQAS was 100%. The NBTS has been certified ISO2008-9001 and nucleic acid testing (NAT) has been introduced in the routine screening of blood donations for TTIs. Mauritius has national guidelines on the appropriate clinical use of blood and blood products and has established a nonpunitive haemovigilance system to reduce and eliminate the adverse reactions associated with blood transfusion in both donors and recipients. A national haemovigilance committee has been set up, focal haemovigilance officers were appointed, and adverse reaction reporting forms were printed and sent to all hospitals for gathering of data. All blood transfusion activities performed at the NBTS and the management of information have been fully computerized.

Total NBTS personnel grew from 56 employees in 2002 to 82 in 2016, with a medical-technical staff including 1 physician, 15 nurses, 15 health care assistants, 22 laboratory technicians, 8 laboratory assistants, and 6 blood bank assistants, whose number increased gradually from 44 (78.5%) in 2002 to 69 (84.1%) in 2016. The other personnel include motivators, general workers, and drivers. The full list, qualifications, and organogram are available at the NBTS. The NBTS has a continuing education programme for personnel involved in blood transfusion, but there is no training programme that offers a nationally recognized university degree or diploma in blood transfusion as a medical science.

The annual budget of NBTS is allocated by MoH&QL. The average amount of the allocated budget earmarked for blood safety is 2,450,000 USD. Furthermore, the country has set up a partial cost recovery system targeting private health institutions. The approximate unit cost of one unit of whole blood and Red Cell Concentrate (RCC) unit has been estimated at 42 USD. Components such as platelets, fresh frozen plasma (FFP), and cryoprecipitate were provided at an additional cost of 15 USD each, which gave a unit cost of 55 USD. There is no external funding for blood safety in the country.

### 3.2. Blood Donations

Over the period, a total of 525,700 units of blood were collected in the country from 2002 to 2016. The total number of blood units collected per year increased from 31,228 in 2002 to 43,742 in 2016 with a peak of 49,349 in 2013.

**Figure 1:**
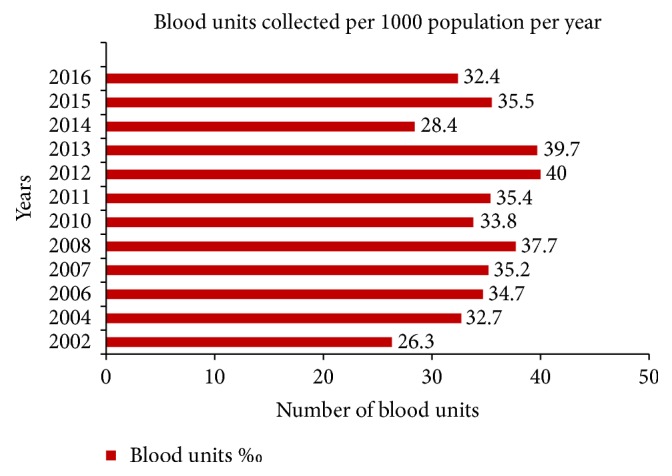
Blood donation rate per 1000 population per year in NBTS of Mauritius from 2002 to 2016.

The average annual blood donation rate per 1,000 inhabitants was 34.3 units, having grown from 26.3 units in 2002 to 32.4 units in 2016, with a peak of 40.0 in 2012 (Figure 1).

The percentage of VNRBD rose from 60% in 2002 to 86.2% in 2016, with slight peaks of 90.8% in 2014 and 92.4% in 2015 and an average percentage of 82.5%.

Furthermore, as one of the activities of the 2015 World Blood Donor Day, blood donor association of Mauritius (BDAM) jointly organized with the NBTS an Africa and Asia Pacific meeting for blood donor motivation. Donor officers/recruiters/educators from African NBTS and members of blood donors' associations or other relevant stakeholders in African countries were invited to attend this important meeting. The cost of participation and three-night stay was borne by BDAM.

Since 2010, the NBTS in Mauritius performs aphaeresis donations. The numbers of blood donations collected using aphaeresis were 60 (0.1%) in 2010, 131 (0.3%) in 2011, 117 (0.2%) in 2012, and 107 (0.2%) in 2013.

The deferral rate of blood donors decreased between 2006 and 2013 from 23.0% to 11.9% with an average rate of 14.7%. Potential blood donors were deferred from donating mainly for low haemoglobin 3.8%, other medical conditions 5.0% (namely, the donation period limit, pregnancy, lactation, and lack of rest), and other reasons 5.7%.

### 3.3. Blood Donation Testing

All donated blood units in the four other regional blood banks are brought to the NBTS at Victoria Hospital Candos for testing before processing, storage, and distribution for use in various hospitals and private clinics.

The following tests are performed on all blood units collected since 2002: tests for TTIs by ELISA prior to the introduction of NAT tests in the routine screening (HIV1 + 2 antibodies and p24 antigen for HIV, HBsAg for HBV, anti-HCV antibodies for HCV, and VDRL and TPHA for syphilis); blood grouping (ABO red cell and serum grouping, rhesus D grouping); and rhesus phenotype and screening tests for irregular blood group antibodies. The average percentage of reactive blood units for TTIs was 0.1% for HIV and HBV, 0.5% for HCV, and 0. 3% for syphilis. These percentages were ranged from 0.1% to 0.4% for HIV, 0.1% to 0.2% for HBV, 0.1% to 1.3% for HCV, and 0.1% to 0.5%.

With regard to blood grouping, the frequency of blood groups in blood donors was as follows: 40.0% for group O, 27.0% for group A, 26.0% for group B, and 7.0% for group AB. About 96.0% of blood donors in Mauritius are rhesus D (RHD) positive and 4.0% are RHD negative.

### 3.4. Blood Components Preparation

The separation of whole blood donations into components is a centralized activity and the following components are prepared from a unit of whole blood: RCC, whole-blood-derived platelet concentrates (WBDP), FFP, recovered plasma, and cryoprecipitate. Moreover, platelets are also prepared through apheresis procedures.

The percentage of blood donations separated into blood components increased gradually from 40.0% in 2002 to 98.2% in 2016. These components are stored in appropriate cold chain equipment for blood (refrigerators, freezer, platelet agitators, etc.). The numbers of various components prepared per year are indicated in Table 1.

Out of 366,198 whole blood donations submitted to separation process between 2008 and 2016, a total of 209,908 units of RCC were prepared, followed by WBDP with 142,849 units, FFP with 125,810 units, and cryoprecipitate with 3,610 units. The remaining 156,290 units of blood units collected were still used as whole blood over the period. Furthermore, 917 units of apheresis platelets were prepared over the period. In 2016, out of 43,742 units of blood collected, 42,949 were separated into red cells.

All blood units found reactive for TTIs are destroyed by incineration. Blood units found safe (i.e., negative for TTIs) are labelled and stored. The label on a blood unit carries the following information: ABO and rhesus group, date of collection, date of expiry, blood unit number, and warning label (product name, volume, status of TTI, etc.).

### 3.5. Clinical Use of Blood, Blood Components, and Blood Products

The NBTS is responsible for supplying blood to all public and private institutions in the whole country. The transport of blood to regional hospitals is centralized and done by the trained staff of NBTS. Appropriate cold boxes are used for transporting blood with temperature monitoring. Blood stocks management including the cold chain maintenance is done on a daily basis and a minimum of 4-day stock is kept. In addition, procedures are in place for cold chain maintenance. Out of 5 regional public hospitals and 22 private health institutions performing blood transfusion, only 1 (3.7%) has a hospital transfusion committee (HTC). All requests for blood, when patients need transfusion, are sent to the compatibility testing department. It is only when the blood unit is compatible that it is issued to the patient. The use of blood and blood products is as follows: medical conditions 21.0%, dialysis 20.0%, surgeries 12.5%, women (obstetrics and gynaecology) 12.2%, radiotherapy/oncology (cancers) 12.0%, orthopaedic 7.0%, cardiac surgery 6.0%, burns 5.3%, and paediatrics/children 4%.  Table 2 indicates some wastage numbers per year of various blood components.

The main adverse reactions reported were nonhaemolytic febrile and mild allergic reactions, but the numbers of cases were indeterminate. In 2011, a case of transfusion associated HBV infection was reported.

The essential medicines list (EML) in the country includes the following plasma-derived medicinal products (PDMP): intravenous immunoglobulin (IVIG), Factor VIII, and Factor IX. However, all these products are imported from abroad.

### 3.6. Other Services Delivery

In addition to blood safety activities, the NBTS has other departments performing specific services for all public and private health institutions, namely, antenatal serology, tissue typing (HLA typing), special investigations, and reagent preparation.

In the antenatal serology department, tests performed on antenatal patients include ABO red cell and serum grouping, rhesus grouping, screening test for irregular blood group antibodies, antibody identification, and antibody titration. The tissue typing department deals with patients and potential donors undergoing renal or bone marrow transplant, as well as the following tests: HLA class I and HLA class II antigens and direct cross-matching for compatibility between donor and recipient. The prospective recipients and donors are also tested for blood groups, HIV, hepatitis B, hepatitis C, and syphilis. The special investigations and reagent preparation departments (red blood cells as reagents, panel for research, identification of irregular antibodies, etc.) carry out investigations on blood group discrepancies, incompatibility due to blood group antibodies, transfusion reaction, antibody screening tests, antibody titration, cold antibodies, cord blood, and red cell phenotyping.

## 4. Discussion

The success of Mauritius in blood transfusion is a result of a strong political commitment leading to an adequate investment and good governance in area. Indeed since 2002, the Government from Mauritius has shown a good will and a great commitment in adhering to WHO principles and priorities for keeping blood related issues among its health strategic goals [1, 3, 10]. Beyond the political and economic reasons that have allowed the progress of the blood safety and availability in the country, other reasons, such as the small size and population of the country, allow the oversight of the centralized blood safety process, in contrast to large countries in the African Region. As a result, Mauritius essentially has a centralized and nationally coordinated blood service within its regional hospital premises. All blood units collected are tested, processed, stored, and distributed for clinical use at the NBTS whose central location in the central Plaines Wilhems district of the country makes it easy to distribute blood to all centres. Furthermore, a project for extension of the current NBTS is underway to create more space for new activities [11]. This entity is different from the multiplicity of uncoordinated blood services facilities found in some Sub-Saharan African countries. This centralized and nationally coordinated blood service has benefits such as consistent standards, harmonization of practices, better utilisation of resources, and being more cost-effective to ensure the safety and quality of products.

However, the haemovigilance system is still in its infancy and although national standards for blood transfusion were developed and established, the absence of the blood regulatory system does not allow ensuring that these standards are met for the production of blood components and monitoring of blood safety, since the NBTS of Mauritius is both manufacturer and regulator of blood and blood products. Indeed, the regulatory oversight aspect should be under the responsibility of a National Regulatory Authority (NRA) and blood regulation additionally addresses efficient management of the national blood system, adequacy of the blood supply, and equitable access to transfusion therapies including PDMP [12].

Providing an adequate number of qualified and trained staff, both medical and paramedical, in the NBTS to carry out blood safety activities is very important. Current trends show that the number of technical staff members of NBTS is growing and it is sufficient. Even though there is only one physician, trained nurses are available to perform predonation counselling maintaining privacy and confidentiality. The continued training programme in place aims to strengthen the capacity of staff involved in blood transfusion services on all aspects of blood transfusion. However, other physicians with a diploma in transfusion medicine should be trained, recruited, and assigned to the NBTS.

With regard to funding of blood safety, the commitment of government to allocate a budget to the NBTS of Mauritius on regular basis combined with the partial cost recovery system established through payments made by private health care facilities for blood units accounts for 100% of the funding for sustainable blood safety and availability in the country. However, in view of technologies and strategies used for collection, testing, processing, storage, and distribution of blood and blood products, the unit cost of a blood unit for transfusion seems low.

The numbers of the VNRBD and the blood donations rate per 1000 population in Mauritius have been steadily rising. Collecting blood through VNRBD from a low-risk population is a WHO recommendation for ensuring blood safety. Since 2007, Mauritius has reached the target defined by the regional strategy for blood safety, which urges Member States to collect at least 80% of their blood donations from VNRBD [3, 8, 13]. Furthermore, from 2002 to 2016, Mauritius has achieved almost the same blood donation rate as high-income countries, which is 36.8 donations per 1000 population [14, 15]. The increasing of blood donations rate per 1000 population indicates the country made effort for blood donations in order to meet the annual requirements for blood and blood components. This is not the case for some big countries like Nigeria where blood donations per 1000 population and further highlight a very poor blood supply chain in the country [16]. Indeed, in order to increase blood donations, the NBTS collects blood at fixed points and through a mobile blood collection system throughout the island with the assistance of civil society, the Blood Donors Association of Mauritius (BDAM) registered in 1999, the association of blood donation organizers, and other nongovernmental organizations (NGOs). The fixed blood collection points are located at the blood banks attached to the four regional hospitals. The NBTS provides logistical support to blood collection activities. Blood donor recruitment involves education, information, and motivation techniques which ultimately convert nondonors into voluntary blood donors [17].

Even though, the deferral rate of blood donors in Mauritius decreased from 23% to 11.9% between 2006 and 2013, the current deferral rate is almost the same as the one found in a multicentric study in Sub-Saharan Africa but remains slightly higher than the average of the deferral rate of 8.6% observed in Namibia in 2016 [18, 19].

Regarding the blood testing, the percentages of blood units reactive for TTI markers in blood donors were decreasing over the past fifteen years. The improvement of the safety and quality of blood and blood components in the country is due to huge efforts made by the NBTS in the implementation of a quality management system through the development of standards and guidelines for blood safety and its participation in the EQAS for TTI testing, blood grouping, antibody screening and identification, and compatibility testing. In addition, the certification ISO9001:2008 of the NBTS in 2010 is an important step towards accreditation. Furthermore, the introduction of automation in blood grouping and TTI screening with interfacing of the equipment with computer systems has further enhanced blood safety by eliminating manual errors. The NAT introduced in the routine practice is an advanced screening technology that reduces the window period of TTIs and helps to improve blood safety. It is a method which screens the genetic material of the disease causing organisms which can detect the infection faster than the conventional serology tests. Indeed, it detects HIV-1 within 4.7 days after transmission, HCV within 2.2 days as against relatively very longer period through the conventional ELISA [20]. Indeed, Mauritius is one of the three countries (including South Africa and Namibia) out of 47 in the African Region which have introduced NAT in the routine screening of blood donations [13].

The seroprevalence of TTI markers in Mauritius blood donors observed during the past fifteen years, especially since 2008, is very low, suggesting the low residual risk of TTIs in blood donors and a low risk of transmission of bloodborne infections to blood recipients. Reliance on VNRBD at lower risk for TTIs combined with quality-assured laboratory screening with highly sensitive assays provides additional safety, compared to the 2006 and 2010 survey and other study findings that these requirements are not often met in the most countries in the African Region [8, 21]. Indeed, it has been proven in other studies in Africa that stringent donor selection criteria and improved screening technologies have reduced the number of TTI-positive donors screened during donation and improved the detection of TTIs among donors [22].

The phenotypic frequency of RH D positive and RHD negative in blood donors in Mauritius is close to the frequency observed in Sub-Saharan Africa [23–25]. As a result, there is often a shortage of RHD negative blood. To address this issue, the NBTS maintains a list of these donors so that they can be called upon when required.

With regard to processing of blood donations, the number of blood components prepared from a unit of whole blood (RCC, whole-blood-derived platelet concentrates, apheresis platelet, FFP, and cryoprecipitate) is an indicator that each transfused patient receives the blood component he/she needs. There has been a significant increase in the proportion of whole blood units separated into components over the years from 40% in 2002 to 98.2% in 2016. Due to the low seroprevalence of TTIs in blood donations, the proportion of blood units discarded for these infections remains very low. This further underscores the point that collecting blood from VNRBD remains the foundation of any safe blood supply system in a country.

The percentage of blood components issued and transfused has increased in the country over the years, especially RCC, whole-blood-derived platelet concentrates, and FFP. Taking into account the ordering and delivery, the blood stocks in all hospitals and NBTS are monitored daily and blood is equitably distributed based on existing stocks and anticipated needs. According to the MOH&QL in Mauritius, the demand for blood continues to grow each year, possibly due to the expansion in health services; improved health care facilities; the introduction of sophisticated medical procedures such as renal transplant and cardiac surgery; and the increased number of cancer patients who need transfusion support during chemotherapy [26]. Accidents and trauma generally account for massive transfusion in the country. Unlike the situation in mainland Africa where the burden of infectious diseases is still high, noncommunicable diseases (NCD) such as diabetes and cardiovascular diseases are emerging as a major challenge for Mauritius [26]. Until recently, 30% of the blood supply was used to treat anaemia in end-stage and renal failure patients, whereas in the most of Sub-Saharan Africa countries blood supplies are mainly used for severe obstetric haemorrhage, severe anaemia associate with malaria infection, surgery and trauma, and sickle cell disease [26, 27]. The overall reduction of the proportion of transfused whole blood stems from the improved separation of whole blood into components. The NBTS has also played a crucial role in advocating for the introduction of blood alternatives where possible; one example is the introduction of Erythropoietin (EPO), rather than blood transfusion, to treat kidney anaemia, thus reducing the use of blood by renal dialysis units from 30% to almost 10%. However, the practice on the use of EPO in Sub-Saharan African patients is relatively rare [16, 28].

One of the major challenges of NBTS has been a weak clinical interface for transfusion, evident in the very limited proportion (3.7%) of hospitals that have established a hospital transfusion committee (HTC). Although a haemovigilance system is in place, implementation is fragmented and there is underreporting of transfusion-related adverse reactions. This is largely attributed to the absence of HTCs. The haemovigilance system is still implemented and the reported adverse reactions appear to be underestimated since they are based on reporting from clinical units that may not register or send all cases to the blood services. The proportion of patients transfused according to age and clinical situation was not recorded. Mauritius, like 19 other countries out of 47 in the WHO African Region, has included PDMPs on its essential medicines list and imports these products from abroad [13].

The existence of other services performing specific immunohematological analyses, HLA phenotyping and reagents preparation, has transformed the NBTS in Mauritius into a comprehensive service for both donors and patients. Indeed, most of the NBTS in the region are only focused on analyses for the qualification of blood donations and the delivery of blood for transfusion to recipients.

The example of Mauritius shows that it is possible to make progress for blood safety and availability in African countries. This progress mainly needs to carry out a situation analysis in order to develop the national blood policy and the strategic plan for its implementation. The national blood policy should define the goal and objectives, strategic orientations, and the implementation framework, while the strategic plan should define vision and mission, objectives, priory interventions with logical frameworks indicating, on the one hand, the strategic objectives, expected results, and indicators and, on the other hand, the expected results, priority interventions, implementing bodies, timeframe, and cost. The implementation of both requires an efficient organizational structure, adequate qualified and trained staff, and a financial sustainability including the development of a national health financing strategy based on a vision where all populations have access to quality health respecting the equity and solidarity principles.

## 5. Conclusions

Over the past fifteen years, Mauritius has made commendable efforts to ensure the availability and accessibility of safe and quality blood and blood products. Indeed, with the good will and commitment from its government and policy makers, reasonably safe blood can be a reality in the African context. The NBTS has overcome many challenges in terms of voluntary low-risk blood donors, quality blood testing, quality service delivery of blood for transfused patients nationwide, and specialized service delivery in antenatal serology, tissue typing, special investigations, and reagent preparation. Although Mauritius has made progress in addressing blood safety issues, some challenges still remain and include the lack of transfusion legislation, a weak national regulatory system for blood and blood products, no production cost per unit of blood intended for transfusion, and a weak clinical interface for transfusion.

These challenges call for concrete and appropriate actions such as (i) strengthening of the legal framework through the enactment of transfusion legislation and the establishment of a national regulatory system for blood and blood products; (ii) conducting a costing study as a management tool for monitoring NBTS costs; (iii) setting up an appropriate mechanism for data collection and information management in NBTS; (iv) enhancing the establishment of HTCs and other mechanisms for reporting adverse reactions and linkage between hospitals and blood transfusion centres; and (v) contracting fractionation into PDMP with plasma fractionators in order to meet national demand for these products given the high quality of the plasma produced.

## Figures and Tables

**Table 1:** Numbers of blood components prepared from 2002 to 2016.

Year	RCC	PLT whole blood derived	Apheresis PLT	FFP	Cryoprecipitate
2002	NA				
2004	16,122				
2006	23,936				
2007	9,336				
2008	13,426	12,825	122	12,839	609
2010	20,894	11,772	44		
2011	18,902	10,408	117	11,495	235
2012	20,944	10,514	107	12,105	334
2013	24,625	17,437	100	13,819	485
2014	37,385	27,451	104	24,835	595
2015	44,209	27,486	88	25,816	759
2016	42,949	24,956	235	24,901	593

**Table 2:** Wastage numbers of blood components in Mauritius from 2010 to 2016.

Year	Red cells/whole blood	Platelets	Plasma
2010	1,678	372	422
2011	1,238	1,714	435
2012	1,775	2,280	391
2013	2,209	2,130	646
2014	2,362	2,788	2,013
2015	2,427	3,404	1,282
2016	1,564	2,981	1,670
